# Social participation and coronary heart disease risk in a large prospective study of UK women

**DOI:** 10.1177/2047487315607056

**Published:** 2015-09-28

**Authors:** Sarah Floud, Angela Balkwill, Dexter Canoy, Gillian K Reeves, Jane Green, Valerie Beral, Benjamin J Cairns, for the Million Women Study Collaborators

**Affiliations:** Nuffield Department of Population Health, University of Oxford, UK

**Keywords:** Coronary heart disease, incidence, social participation, women

## Abstract

**Background:**

Participation in social activities is thought to prevent heart disease, but evidence is inconclusive.

**Design:**

We assessed whether participating in social activities reduces the risk of coronary heart disease (CHD) in a large prospective study of 735,159 middle-aged UK women.

**Methods:**

Women reported their participation in eight social activities (religious group, voluntary work, adult education, art/craft/music, dancing, sports club, yoga, bingo) and were followed for first CHD event (hospital admission or death) over the next 8.6 years. Cox regression models were used to estimate relative risks for CHD incidence by participation in each and in any of the social activities.

**Results:**

After adjustment for age and region only, every activity except bingo was associated with a reduced risk of CHD (*n* = 30,756 cases in total). However, after additional adjustment for 11 factors (deprivation, education, smoking, physical activity, body mass index, alcohol, marital status, self-rated health, happiness, hypertension, diabetes), every relative risk estimate moved close to 1.0. For example, for participation in any of the activities compared with none, the relative risk adjusted for age and region only was 0.83 (99% confidence interval 0.81–0.86), but changed to 1.06 (99% confidence interval 1.02–1.09) after additional adjustment. Adjustment for education, self-rated health, smoking and physical activity attenuated the associations most strongly. Residual confounding and other unmeasured factors may well account for any small remaining associations.

**Conclusions:**

Associations between participation in various social activities and CHD risk appear to be largely or wholly due to confounding by personal characteristics of the participants.

## Introduction

It has been suggested that social participation might prevent heart disease^1–3^ but evidence is sparse and inconclusive.^4,5^ Almost all previous studies of social participation and health^4–10^ have grouped together substantively different activities, such as attending religious services, attending cultural events, and being a member of a sports club, each of which might be related to different socio-economic and lifestyle profiles, which in turn might affect associations with health in different ways. Furthermore, there is potential for reverse causation bias, whereby poor health might deter, or sometimes encourage, participation in certain activities, and few studies have investigated this possible source of bias. In a large prospective cohort of women in the United Kingdom, we investigated the association of participation in eight types of social activity with the risk of a first coronary heart disease (CHD) event. For each activity, we examined associations with CHD risk adjusted for age and region of residence only, the effect of additional adjustment for 11 socio-economic and lifestyle factors, and the extent to which associations might be biased due to reverse causation.

## Methods

### Participants

The Million Women Study is a population-based prospective study of 1.3 million middle-aged women recruited in 1996–2001 through the UK National Breast Screening Programme.^11^ At recruitment, women self-completed a health and lifestyle questionnaire, and gave written consent to participate. Study questionnaires and further details of the data and access policies can be viewed on the website (www.millionwomenstudy.org). Ethical approval was provided by the Oxford and Anglia Multi-Centre Research Ethics Committee. About three years after recruitment, women were sent a further postal questionnaire and were asked for the first time about participation in social activities (65% response rate). This was the baseline for the present study.

Individuals in the study are linked by their unique National Health Service (NHS) number to NHS Central Registers, through which they are followed up for death, emigration and cancer registration, and to NHS hospital admissions databases. Follow-up for hospitalizations and deaths is 99% complete, as only 18,970 (1%) have been lost to follow-up in the entire cohort. Dates of hospital admissions and diagnoses, coded to the World Health Organization’s International Classification of Diseases 10th revision (ICD-10), were obtained by electronic record linkage to the Hospital Episode Statistics for England and the Scottish Morbidity Records in Scotland. Women were followed until 31 March 2011 in England (31 December 2008 in Scotland) because hospital admission data were incomplete after these dates.

### Definitions

Participation in social activities at baseline was assessed by asking the women if they belonged to or participated in any of the following: ‘religious group’, ‘voluntary work’, ‘sports club’, ‘dancing group’, ‘yoga’, ‘art/craft group’, ‘music/singing group’ and ‘bingo’. ‘Art/craft group’ and ‘music/singing group’ were combined into one variable called ‘art/craft/music’.

The outcome for these analyses is the first CHD event (any hospital admission with a diagnosis of CHD, or death with CHD as the underlying cause, whichever came first, coded as ICD-10 I20-I25). In a sample of hospital admissions, 92% of diagnoses coded to ICD-10 I20-I25 were confirmed as CHD by primary care physicians.^12^

### Analysis

A total of 866,341 women completed the baseline questionnaire. We excluded 73,894 (8.5%) who reported heart disease or stroke at recruitment or at study baseline (or had been admitted to hospital for these conditions prior to baseline), and another 42,354 (4.9%) who had a previous cancer registration (except non-melanoma skin cancer), because these conditions might affect both participation in social activities and CHD risk. A further 14,934 (1.7%) women were excluded because they were not asked about bingo. The remaining 735,159 women formed the population at risk for these analyses.

Correlations between social activities were assessed using Spearman’s rank correlation. Questions about the same activities were repeated in a subsequent resurvey, four years after baseline (mean 4.4 years (SD 1.2)); agreement was assessed using kappa statistics.

We used Cox regression models to estimate hazard ratios (referred to hereafter as relative risks (RRs)) of a first CHD event in relation to participation in different social activities, using attained age as the underlying time variable. Owing to the large number of relative risks estimated, results are given with 99% confidence intervals (CIs). The date when women reported their participation in social activities is the baseline date for these analyses. Person-years were calculated from this baseline until the date of hospital admission for CHD, death, emigration or end of follow-up, whichever came first.

The regression models were stratified by region of recruitment (10 regions). Then, adjustment was made for 11 additional variables, separately and together. The variables were: deprivation in fifths, based on the Townsend Index, a score incorporating census area data for employment, car ownership, housing tenure and household overcrowding;^13^ education (tertiary qualifications (college/university), secondary qualifications (A levels/O levels, usually obtained at 18 and 16 years old respectively), technical qualifications (nursing, teaching, clerical or commercial), completed compulsory schooling with no qualifications, did not complete compulsory schooling); marital status (married or living with a partner, not married or living with a partner); cigarette smoking (never, past, current <15 per day, current ≥15 per day); alcohol intake (0, <7, 7–14, ≥15 units per week); physical activity (rarely or never, <once per week, ≥once per week); body mass index (BMI) (<22.5, 22.5–24.9, 25.0–27.4, 27.5–29.9, ≥30 kg/m^2^); self-rated health (excellent, good, fair, poor); happiness (most of the time, usually, sometimes, rarely/never); treatment for hypertension (yes/no) and treatment for diabetes (yes/no). Missing values of the adjustment variables (<3% for each variable) were assigned a separate category. Information on all variables was self-reported at baseline except for education and physical activity, and height in the calculation of BMI, which were self-reported at recruitment, and deprivation, which was calculated using the postcodes of respondents at recruitment.

The evidence of association after adjustment for the above factors was indicated by the likelihood-ratio χ^2^ statistic. If the χ^2^ value was substantially reduced after adjustment (e.g. by more than 50%), it is plausible that any remaining association is, at least in part, due to residual confounding, because of potential measurement errors in the adjustment variables.^14^ We investigated potential for proportional hazards violations by testing for heterogeneity by age at diagnosis, but there was no evidence against the proportional hazards assumption for any of the activities.

We addressed possible reverse causation bias in two ways, firstly by adjusting for self-rated health reported at baseline, and also by repeating the main analysis excluding the first four years of follow-up.

We conducted analyses in subgroups of women and tested for heterogeneity in the main associations using a χ^2^ contrast test. We also investigated whether participation in each specific activity might lead to changes in behaviour, by comparing the prevalence of smoking, BMI and alcohol consumption at baseline and four years later in women who were not participating in each activity at baseline but who were participating in the activity four years later, and also, for comparison, in women who were participating in each activity at baseline but not four years later.

All analyses used Stata 13.1 (StataCorp., College Station, TX, USA).

## Results

The 735,159 women without prior heart disease, stroke or cancer included in these analyses had a mean age of 59.8 years (SD 4.9) at baseline. Almost two-thirds (59%) reported taking part in at least one social activity, with a mean of 1.7 (SD 1.0) activities for those who engaged in at least one. Table 1 shows the characteristics of women included in the analysis, by type of social activity. Sports club and voluntary work were the most common (about 20% reporting participation) and the least common were dancing, yoga and bingo (about 7% reporting participation). Compared with women not participating in any social activity, participants in each specific activity, except bingo, tended to be more educated, to be of a higher socio-economic position, to report healthier behaviours (i.e. not smoking, not being overweight, and engaging in physical activity) and to be less likely to rate their health as poor. By contrast, women who participated in bingo were less educated, of a lower socio-economic position and were more likely to be current smokers, overweight, physically inactive and to rate their health as poor.

**Table 1.** Baseline characteristics of women by type of social activity.

	Type of social activity								
	Religious group	Voluntary work	Adult education	Art/craft/music	Dancing	Sports club	Yoga	Bingo	None of these eight activities
*N* (%)	129,206 (18)	139,383 (19)	89,421 (12)	95,276 (13)	54,644 (7)	147,125 (20)	51,648 (7)	42,758 (6)	304,474 (41)
Mean no. of social activities (SD)	2.3 (1.1)	2.3 (1.1)	2.5 (1.2)	2.5 (1.2)	2.2 (1.2)	2.0 (1.1)	2.4 (1.2)	1.6 (0.9)	0
Mean age, years (SD)	60.6 (5.0)	60.7 (4.9)	59.9 (4.9)	60.7 (4.9)	60.5 (4.9)	59.7 (4.8)	59.4 (4.7)	60.4 (4.9)	59.4 (4.8)
Most deprived fifth (at recruitment), %	12	11	11	10	14	10	10	37	19
No educational qualifications (at recruitment), %	21	20	13	19	35	24	18	71	49
Not married/cohabiting, %	22	22	23	21	21	17	19	23	18
Current smoker, %	5	7	7	6	8	7	6	28	17
Mean alcohol units/week (SD)	3.8 (5.2)	4.7 (5.7)	5.2 (5.9)	4.5 (5.6)	4.2 (5.1)	5.7 (6.0)	5.6 (5.8)	3.5 (5.3)	4.2 (5.9)
Mean body mass index, kg/m^2^ (SD)	25.8 (4.4)	25.8 (4.4)	25.6 (4.3)	25.9 (4.4)	25.2 (3.8)	25.5 (4.0)	24.5 (3.5)	27.7 (5.1)	26.3 (4.7)
Physical activity (at recruitment), rarely/never, %	38	33	30	33	23	21	21	62	56
Self-rated health, fair/poor, %	17	15	15	16	13	11	11	34	25
Happy, rarely/never/sometimes, %	14	14	17	14	13	13	16	18	20
Treatment for hypertension, %	19	19	17	19	17	17	15	25	22
Treatment for diabetes, %	3	2	2	2	2	2	1	5	3
**Follow-up**									
Number of first CHD events	5120	5386	3118	3622	1987	4754	1379	3016	13,677
Person-years (1000s)	1104	1202	778	821	471	1276	440	354	2615

CHD: coronary heart disease

The different social activities were weakly correlated with each other (see Table S1 in the Supplementary Material online). Religious groups and voluntary work were the most strongly correlated (Spearman’s correlation = 0.28), while bingo was negatively correlated with most other social activities. There was good agreement for most of the activities between participation reported at baseline and four years later, the strongest agreement being for belonging to a religious group (see Table S2 online).

**Figure 1.**
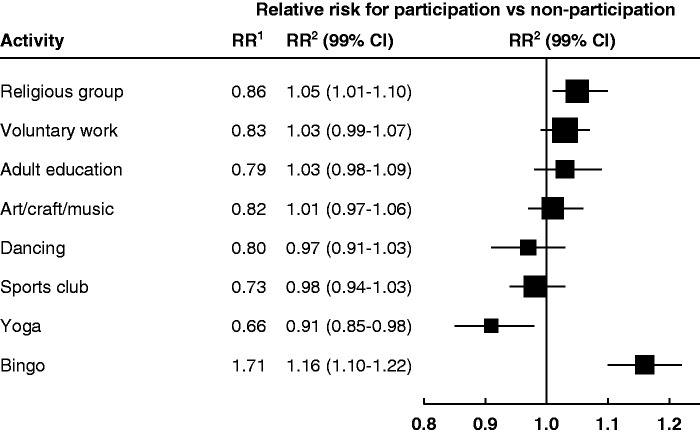
Relative risks for first coronary heart disease event in relation to participation in specific social activities. RR^1^: relative risks stratified by age and region of residence; RR^2^: relative risks stratified by age and region of residence and adjusted for deprivation, education, marital status, smoking, alcohol intake, body mass index, physical activity, self-rated health, happiness, treatment for hypertension, treatment for diabetes. (See Table S3 online for effect of adjustment for each factor separately). CI: confidence interval.

During follow-up for a mean of 8.6 years (SD 2.0), there were 30,756 women with a first CHD event, including 1385 where death from CHD was the first coronary event. With adjustment for age and region only, participation in each social activity except bingo was associated with a lower risk of CHD than not participating in that activity (Figure 1). After adjustment for an additional 11 factors (deprivation, education, marital status, smoking, alcohol intake, BMI, physical activity, self-rated health, happiness, hypertension and diabetes) the statistical strength of every association was substantially weakened, with every risk estimate moving close to 1.0. Overall, compared with women who participated in no activities, those who participated in any activity had a relative risk for CHD of 0.83 (99% CI 0.81–0.86) after crude adjustment for age and region of residence only, but this changed to 1.06 (99% CI 1.02–1.09) after additional adjustment for the 11 other factors (Table 2). For participation in any activity except bingo, the relative risk was 0.75 (99% CI 0.72–0.77) and changed to 1.03 (99% CI 0.99–1.06) after adjustment.

**Table 2.** Relative risks for first CHD event for participation versus non-participation in any of the activities.

	Any activity	χ^2^
	RR (99% CI)	
Adjustment for age and region only	0.83 (0.81–0.86)	255
Additional single adjustment for:		
Deprivation	0.86 (0.84–0.89)	163
Education	0.92 (0.89–0.95)	49
Marital status	0.83 (0.80–0.85)	263
Smoking	0.89 (0.86–0.92)	102
Alcohol	0.86 (0.83–0.88)	174
Body mass index	0.85 (0.83–0.88)	185
Physical activity	0.89 (0.86–0.92)	98
Self-rated health	0.92 (0.89–0.95)	49
Happiness	0.85 (0.83–0.88)	194
Treatment for hypertension	0.85 (0.83–0.88)	184
Treatment for diabetes	0.84 (0.82–0.87)	218
**Adjustment for all of above**	**1.06 (1.02–1.09)**	**20**

There were 17,079 first CHD events among women who participated in any of the eight activities, and 13,677 first CHD events among women who did not participate in any of the eight activities.

RR: relative risk; CI: confidence interval; CHD: coronary heart disease.

Adjustment for education, self-rated health, smoking and physical activity had the greatest effects on the association between participation in any activity and CHD, with each of these factors reducing the χ^2^ value by more than 60% (Table 2). Similarly, adjustment for these four factors had the greatest influence on the associations for most of the specific activities: separate adjustment for education, for self-rated health or for smoking typically reduced the χ^2^ values by 40% or more, and adjustment for physical activity reduced the χ^2^ value by 40% or more for dancing, sports club membership and yoga (see Table S3 online). Similar results were also obtained when women were classified as having participated in no activities, one activity or more than one activity (see Table S4 online).

Across subgroups of women with different smoking patterns and other characteristics there was little variation in the RRs associated with each activity (see Figure S1 online). There was some variation in the risk of CHD in relation to bingo by level of education (*p* = 0.001), but this could be due to chance, given the number of statistical tests conducted.

We assessed the possible role of reverse causation bias in two ways. First we adjusted for self-rated health reported at baseline, and this substantially attenuated the age- and region-adjusted associations for every activity. Second, we excluded the first four years of follow-up, after which the RR estimates were further attenuated (see Table S5 online). Adjustment for treatment for hypertension and diabetes did not make a material difference to the relative risks (see Table S3 online).

Since social participation might lead to changes in some lifestyle factors such as smoking, weight or alcohol consumption, we compared the prevalence of these factors at baseline with the prevalence four years later, both in women who had taken up the activity and in women who had ceased the activity during the four year period (see Table S6 online). In every group, regardless of whether women had taken up or ceased a particular activity, their smoking prevalence declined during the next four years and mean BMI and alcohol intake increased slightly. There was, however, little to suggest greater changes in the prevalence of these factors among women who had started a new activity than among those who had stopped it.

## Discussion

Participation in social activities has been suggested to have a direct effect on the risk of heart disease,^15^ and current UK public health guidelines recommend social participation to prevent the health effects of social isolation.^2^ In this large prospective study of UK women, participation in eight specific social activities and in any of the eight activities combined appeared to have little, if any, direct effect on CHD risk. While each activity except bingo was associated with a lower risk of CHD in crude analyses, after additional adjustment for 11 personal characteristics all the relative risk estimates moved close to 1.0. Adjustment for education, self-rated health, smoking and physical activity resulted in the greatest attenuation of the associations. Participation in every social activity, except bingo, was associated with having educational qualifications, not smoking, being physically active and having good self-rated health, consistent with other evidence on social participation in the UK.^16–18^ In contrast, women who played bingo were more likely to have no educational qualifications, to smoke, to be physically inactive and to have poor self-rated health. That bingo was the only activity associated with an increased risk of CHD argues strongly that any observed association is likely to be largely due to confounding and that social participation has little or no direct effect on CHD risk.

Previous prospective studies that investigated the role of social activities on health have usually grouped together different types of activities.^6–10^ Religious activity is an exception, however, and its association with health has been investigated, but mostly for outcomes other than CHD.^19^ We are aware of only one cohort study of religious activity with CHD as the outcome, and our finding of little or no relation of CHD risk with religious activities agrees with results from that study.^20^ The benefits of engaging in leisure-time physical activity for reducing CHD risk are well-documented.^21,22^ We have reported that physical activity reduces CHD risk in this cohort^23^ and our present findings suggest that it is the physical activity itself, rather than a direct effect of social participation in groups doing physical activity, that accounts for the lower risk of CHD. As far as we are aware there have been no prospective investigations of the associations between CHD risk and the other types of activities we examined. When social activities are grouped together, the evidence in relation to CHD risk is limited. A significant association between a social participation index and CHD risk was reported in a Swedish cohort study, but the risk estimates were not adjusted for physical activity or self-rated health.^4^ Another prospective study in Sweden found an association between a social participation index and myocardial infarction which was attenuated after adjustment for lifestyle factors and self-rated health, but there was uncertainty in the estimates due to there being fewer than 200 cases.^5^

We adjusted for education and deprivation, because more educated women and those of higher socio-economic status are likely to have the resources to pursue social activities.^18^ We also adjusted for marital status and happiness, as social participation might reduce stress caused by social isolation, but these variables were not strong confounders and had comparatively little effect on the RR estimates.^4,24–26^ Poor health is an important potential confounder, as it may affect social participation and also CHD risk.^27^ Although we excluded women with known prior heart disease, stroke and cancer from the analyses, participants in each activity except bingo were less likely to report that their health was poor, and adjustment for self-rated health had strong attenuating effects on associations with CHD risk.

It has been proposed that lifestyle factors may mediate the relationship between social participation and CHD through the social influence of other group members on health-related behaviours, leading, for example, to cessation of smoking and maintenance of a healthy weight,^24,28,29^ although membership of some groups might reinforce unhealthy behaviours.^30^ However, when we looked at changes in some of these lifestyle factors over a four year period, comparing women who took up a new social activity and women who ceased the same activity, we found little discernible change in behaviour associated with changes in participation. We did find, however, that the prevalence of smoking decreased and that mean BMI and alcohol intake increased in all groups, reflecting national trends.^31^ This evidence, albeit weak, suggests that these behaviours are unlikely to be important mediators of CHD risk (i.e. social participation does not appear to have a major effect on CHD risk by altering behaviour).

There are limitations to consider in the interpretation of the results. Women were not asked about every possible activity, and our measure of exposure does not capture how frequently the women participated in each social activity, but this should not bias the results. Participation in different activities can change over time, although evidence in this cohort suggests that changes were not great during the 4-year period after baseline, the most stable activity being participation in religious groups. Little information was collected on lifestyle factors before middle-age or on lipids, which could have resulted in some residual confounding. Serum cholesterol was measured on only a small sample of the population, but we have information on use of statins and adjusting for this altered the RRs by 0.02 or less. These findings are likely to be generalizable to middle-aged UK women, since at recruitment the participants represented one in four UK women in the target age range.

Strengths of the study include its large size, the availability of information on many potential confounding factors, virtually complete follow-up and the demonstrated reliability of the main outcome measure.^12^ Also by examining associations with specific types of social activities, we could show that the activities associated with favourable characteristics have different associations with CHD risk than the activity (bingo) associated with unfavourable characteristics.

In conclusion, crude associations suggesting lower risks of CHD among women participating in various social activities appear to be largely or wholly due to confounding by factors such as education, smoking, physical activity and self-rated health. Hence, in groups of women who are otherwise similar with respect to their socio-demographic, lifestyle and general health, taking part in social activities appears to have little or no material effect on CHD risk.

## Supplementary Material

Supplementary material
