# Supplementary material for: Social participation and coronary heart disease risk in a large prospective study of UK women
Source: Eur J Prev Cardiol. 2015 Sep 28;23(9):995–1002. doi: 10.1177/2047487315607056 (PMC4871172; doi:10.1177/2047487315607056)
Supplement: Supplementary material [file 150428_Social_Participation_online_only_material_607056.pdf]

Web Supplement for:

Social participation and coronary heart disease risk in a large prospective study of UK women

Sarah Floud; Angela Balkwill; Dexter Canoy; Gillian K Reeves; Jane Green; Valerie Beral, and Benjamin J Cairns, for the Million Women Study Collaborators

Cancer Epidemiology Unit, Nuffield Department of Population Health, University of Oxford, Oxford, UK (SF, AB, DC, GKR, JG, VB, BJC)

Table of contents

Table S1. Correlations between different social activities 2

Table S2. Agreement of reported participation in social activities between baseline and four years later 3

Table S3. Relative risks for first CHD event in relation to participation in specific activities, showing effect of each single adjustment 4

Table S4. Relative risks for first CHD event in relation to participation in one or more activity compared to participating in none of the eight activities 5

Figure S1. Relative risks for first CHD event in relation to participation in specific activities, by subgroup 6

Table S5. Relative risks for first CHD event in relation to participation in specific activities, excluding the first four years of follow-up 7

Table S6. Lifestyle factors at baseline and four years later in women who changed participation during the four years 8

Acknowledgements 9

Table S1. Correlations between different social activities

**Social activity**	Religious group	Voluntary work	Adult education	Art/craft/ music	Dancing	Sports club	Yoga	Bingo
Religious group	1.00							
Voluntary work	0.28	1.00						
Adult education	0.09	0.15	1.00					
Art/craft/music	0.20	0.16	0.20	1.00				
Dancing	0.02	0.02	0.03	0.04	1.00			
Sports club	0.02	0.07	0.09	0.05	0.06	1.00		
Yoga	0.02	0.06	0.12	0.06	0.04	0.09	1.00	
Bingo	-0.06	-0.05	-0.05	-0.05	0.02	-0.04	-0.04	1.00

Table S2. Agreement of reported participation in social activities between baseline and four years later

Social activity	Agreement	Kappa
Religious group	91%	0.66
Voluntary work	85%	0.49
Adult education	87%	0.32
Art/craft/music	89%	0.50
Dancing	94%	0.46
Sports club	83%	0.40
Yoga	92%	0.37
Bingo	95%	0.47

Table S3. Relative risks for first CHD event in relation to participation in specific activities, showing effect of each single adjustment

Relative risk (99%CI), adjusted for:	Religious group	χ^2	Voluntary work	χ^2	Adult education	χ^2	Art/craft/music	χ^2
Age and region only	0.86 (0.83-0.90)	94	0.83 (0.79-0.86)	168	0.79 (0.75-0.83)	161	0.82 (0.79-0.86)	125
Additional single adjustment for:								
Deprivation	0.89 (0.86-0.93)	57	0.86 (0.83-0.89)	103	0.82 (0.78-0.86)	110	0.86 (0.82-0.90)	73
Education	0.96 (0.92-1.00)	6	0.93 (0.89-0.97)	22	0.93 (0.89-0.98)	13	0.92 (0.88-0.97)	19
Marital status	0.86 (0.83-0.90)	98	0.82 (0.79-0.85)	177	0.79 (0.75-0.83)	170	0.82 (0.79-0.86)	127
Smoking	0.96 (0.92-1.00)	7	0.88 (0.84-0.91)	78	0.83 (0.79-0.88)	95	0.89 (0.85-0.93)	48
Alcohol	0.85 (0.82-0.89)	113	0.84 (0.81-0.88)	130	0.83 (0.79-0.87)	104	0.84 (0.80-0.88)	100
Body Mass Index	0.88 (0.84-0.91)	76	0.84 (0.81-0.87)	136	0.82 (0.78-0.86)	122	0.83 (0.79-0.87)	114
Physical activity	0.88 (0.85-0.92)	65	0.86 (0.83-0.90)	96	0.84 (0.80-0.88)	90	0.86 (0.82-0.90)	74
Self-rated health	0.91 (0.88-0.95)	38	0.90 (0.87-0.94)	48	0.87 (0.83-0.92)	54	0.88 (0.84-0.92)	54
Happiness	0.88 (0.85-0.92)	69	0.84 (0.81-0.87)	136	0.80 (0.76-0.84)	150	0.84 (0.80-0.88)	102
Treatment for hypertension	0.88 (0.84-0.91)	77	0.84 (0.81-0.88)	135	0.81 (0.78-0.86)	123	0.84 (0.80-0.88)	102
Treatment for diabetes	0.87 (0.84-0.90)	86	0.83 (0.80-0.87)	150	0.81 (0.77-0.85)	137	0.83 (0.80-0.87)	111
**All of above**	**1.05 (1.01-1.10)**	**10**	**1.03 (0.99-1.07)**	**3**	**1.03 (0.98-1.09)**	**3**	**1.01 (0.97-1.06)**	**1**
**All above except hypertension and diabetes**	**1.04 (1.00-1.09)**	**7**	**1.02 (0.98-1.06)**	**2**	**1.02 (0.97-1.07)**	**1**	**1.00 (0.96-1.05)**	**0**

Relative risk (99%CI), adjusted for:	Dancing	χ^2	Sports club	χ^2	Yoga	χ^2	Bingo	χ^2
Age and region only	0.80 (0.75-0.84)	104	0.73 (0.70-0.76)	415	0.66 (0.61-0.71)	264	1.71 (1.63-1.80)	673
Additional single adjustment for:								
Deprivation	0.81 (0.76-0.86)	92	0.77 (0.74-0.80)	283	0.69 (0.64-0.74)	207	1.52 (1.45-1.60)	417
Education	0.80 (0.75-0.85)	102	0.79 (0.76-0.82)	230	0.73 (0.68-0.78)	147	1.49 (1.42-1.57)	376
Marital status	0.79 (0.75-0.84)	106	0.74 (0.71-0.77)	404	0.66 (0.61-0.71)	265	1.70 (1.62-1.78)	658
Smoking	0.84 (0.79-0.89)	61	0.77 (0.74-0.80)	284	0.70 (0.65-0.75)	192	1.48 (1.41-1.56)	365
Alcohol	0.80 (0.76-0.85)	94	0.78 (0.75-0.81)	262	0.69 (0.65-0.75)	194	1.64 (1.56-1.73)	583
Body Mass Index	0.83 (0.79-0.89)	64	0.76 (0.73-0.79)	313	0.72 (0.67-0.77)	162	1.58 (1.51-1.66)	500
Physical activity	0.86 (0.81-0.92)	40	0.81 (0.77-0.84)	184	0.72 (0.67-0.77)	156	1.62 (1.54-1.70)	543
Self-rated health	0.88 (0.83-0.94)	29	0.85 (0.82-0.89)	103	0.75 (0.70-0.81)	114	1.48 (1.41-1.55)	368
Happiness	0.81 (0.76-0.86)	88	0.75 (0.72-0.78)	351	0.66 (0.62-0.71)	250	1.70 (1.62-1.78)	657
Treatment for hypertension	0.82 (0.77-0.87)	81	0.76 (0.73-0.79)	330	0.68 (0.64-0.73)	214	1.66 (1.58-1.75)	608
Treatment for diabetes	0.81 (0.76-0.86)	85	0.75 (0.72-0.78)	357	0.68 (0.63-0.73)	228	1.64 (1.56-1.72)	578
**All of above**	**0.97 (0.91-1.03)**	**2**	**0.98 (0.94-1.03)**	**1**	**0.91 (0.85-0.98)**	**12**	**1.16 (1.10-1.22)**	**51**
**All above except hypertension and diabetes**	**0.96 (0.90-1.02)**	**3**	**0.98 (0.93-1.02)**	**2**	**0.90 (0.84-0.97)**	**14**	**1.16 (1.11-1.23)**	**56**

Table S4. Relative risks for first CHD event in relation to participation in one or more activity compared to participating in none of the eight activities

	**0 activities**	**1 activity**	**2+ activities**	χ^2
Number of cases/non-cases	13677/290797	9664/215524	7415/198082	
	RR (99% CI)	RR (99% CI)	RR (99% CI)	
Adjustment for age and region only	1.00 (-)	0.92 (0.89-0.96)	0.73 (0.71-0.76)	478
Additional single adjustment for:				
Deprivation	1.00 (-)	0.94 (0.91-0.98)	0.77 (0.74-0.80)	328
Education	1.00 (-)	0.97 (0.94-1.01)	0.85 (0.82-0.88)	125
Marital status	1.00 (-)	0.92 (0.89-0.95)	0.73 (0.70-0.76)	491
Smoking	1.00 (-)	0.96 (0.93-0.99)	0.81 (0.78-0.84)	229
Alcohol	1.00 (-)	0.94 (0.91-0.98)	0.77 (0.74-0.79)	358
Body Mass Index	1.00 (-)	0.94 (0.91-0.97)	0.76 (0.74-0.79)	364
Physical activity	1.00 (-)	0.96 (0.93-1.00)	0.80 (0.77-0.83)	238
Self-rated health	1.00 (-)	0.98 (0.95-1.02)	0.85 (0.82-0.88)	138
Happiness	1.00 (-)	0.94 (0.91-0.97)	0.76 (0.73-0.78)	391
Treatment for hypertension	1.00 (-)	0.94 (0.91-0.97)	0.76 (0.73-0.79)	371
Treatment for diabetes	1.00 (-)	0.93 (0.90-0.96)	0.75 (0.72-0.78)	414
**Adjustment for all of above**	1.00 (-)	**1.06 (1.03-1.10)**	**1.05 (1.00-1.09)**	**21**

Figure S1. Relative risks for first CHD event in relation to participation in specific activities, by subgroup.

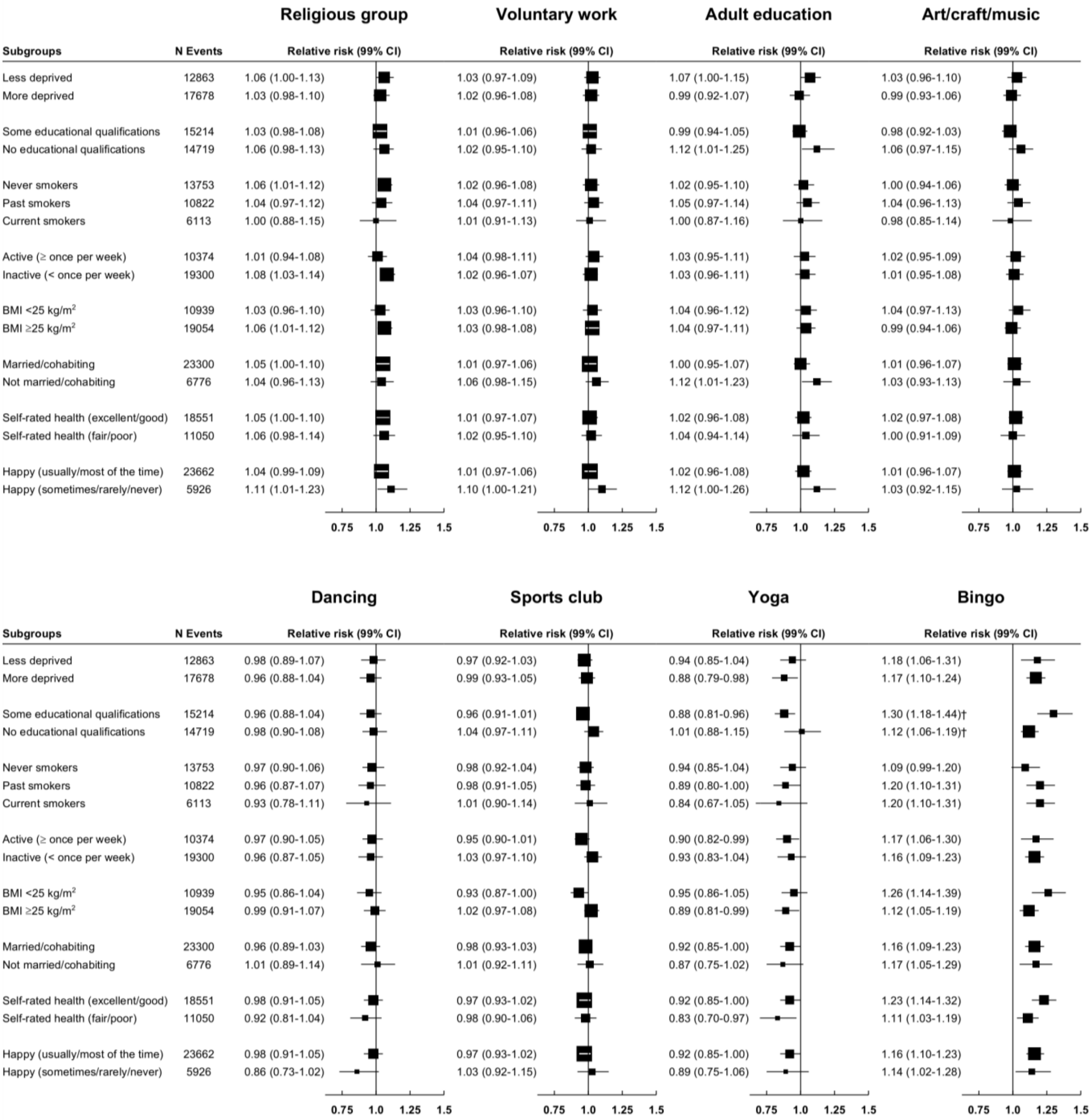

All regression models were stratified by age and region, and adjusted as appropriate for deprivation, education, marital status, smoking status, alcohol intake, body mass index, physical activity, self-rated health, happiness, treatment for hypertension at baseline and treatment for diabetes at baseline.

The differences in risk of CHD between subgroups were assessed using a chi-square test for heterogeneity; † indicates p for heterogeneity <0.01

Table S5. Relative risks for first CHD event in relation to participation in specific activities, excluding the first four years of follow-up

Relative risk (99%CI); N=19,783 first CHD events			
**Religious group**	**Voluntary work**	**Adult education**	**Art/craft/music**
1.04 (0.99-1.10)	1.04 (0.99-1.09)	1.04 (0.98-1.10)	1.02 (0.96-1.08)
**Dancing**	**Sports club**	**Yoga**	**Bingo**
0.97 (0.90-1.04)	1.00 (0.95-1.05)	0.95 (0.87-1.04)	1.14 (1.07-1.22)

All regression models were stratified by age and region, and adjusted for deprivation, education, marital status, smoking status, alcohol intake, body mass index, physical activity, self-rated health, happiness, treatment for hypertension at baseline and treatment for diabetes at baseline.

Table S6. Lifestyle factors at baseline and four years later in women who changed participation during the four years

	Change in participation	N	Current smoker,%		Mean BMI		Mean alcohol units/week	
			at baseline	4y later	at baseline	4y later	at baseline	4y later
**Religious group**	Not participating at baseline, yes 4y later	13567	6.8	4.7	25.8	26.0	4.3	4.7
	Participating at baseline, not 4y later	18342	7.1	5.4	25.9	26.2	4.3	4.7
**Voluntary work**	Not participating at baseline, yes 4y later	49163	7.6	5.5	25.7	26.0	4.9	5.3
	Participating at baseline, not 4y later	28011	8.5	6.4	25.9	26.2	4.6	4.9
**Adult education**	Not participating at baseline, yes 4y later	32961	6.9	4.7	25.6	25.8	5.4	5.8
	Participating at baseline, not 4y later	38154	7.0	5.2	25.7	25.9	5.1	5.5
**Arts/craft/music**	Not participating at baseline, yes 4y later	35226	6.7	4.8	25.9	26.2	4.8	5.1
	Participating at baseline, not 4y later	20828	7.3	5.5	26.0	26.2	4.5	4.9
**Dancing**	Not participating at baseline, yes 4y later	14832	8.1	5.7	25.3	25.5	4.7	5.1
	Participating at baseline, not 4y later	15731	8.2	6.1	25.4	25.8	4.5	4.9
**Sports**	Not participating at baseline, yes 4y later	35347	8.3	5.5	25.9	26.2	5.2	5.6
	Participating at baseline, not 4y later	45500	7.0	5.2	25.7	26.0	5.4	5.7
**Yoga**	Not participating at baseline, yes 4y later	26283	6.4	4.1	24.7	24.9	6.0	6.3
	Participating at baseline, not 4y later	18675	6.1	4.5	24.9	25.2	5.4	5.8
**Bingo**	Not participating at baseline, yes 4y later	10433	19.6	15.0	27.2	27.7	3.7	4.1
	Participating at baseline, not 4y later	7097	23.0	17.6	27.7	28.1	3.6	3.7

The changes in the lifestyle factors over the four years reflect national trends: The Health and Social Care Information Centre. Health Survey for England - 2007, Latest trends. http://www.hscic.gov.uk/2008.

Acknowledgements

The authors thank the women who participated in the Million Women Study as well as the staff from the participating NHS breast cancer screening centres. We also thank the NHS Information Centre for Health and Social Care in England and the Information Services Division in Scotland for the hospital admission data. The figures were prepared with the kind assistance of Mr Adrian Goodill.

The Million Women Study steering committee members are: Emily Banks, Valerie Beral, Ruth English, Jane Green, Julietta Patnick, Richard Peto, Gillian Reeves, Martin Vessey, and Matthew Wallis.

The co-ordinating staff for the Million Women Study are: Hayley Abbiss, Simon Abbott, Naomi Allen, Miranda Armstrong, Krys Baker, Angela Balkwill, Emily Banks, Isobel Barnes, Vicky Benson, Valerie Beral, Judith Black, Kathryn Bradbury, Anna Brown, Benjamin Cairns, Karen Canfell, Dexter Canoy, Barbara Crossley, Dave Ewart, Sarah Ewart, Georgina Fensom, Lee Fletcher, Sarah Floud, Toral Gathani, Laura Gerrard, Adrian Goodill, Jane Green, Lynden Guiver, Michal Hozak, Carol Herman, Isobel Lingard, Sau Wan Kan, Oksana Kirichek, Mary Kroll, Nicky Langston, Bette Liu, Maria-Jose Luque, Kath Moser, Lynn Pank, Kirstin Pirie, Gillian Reeves, Keith Shaw, Emma Sherman, Evie Sherry-Starmer, Helena Strange, Sian Sweetland, Alison Timadjer, Sarah Tipper, Ruth Travis, Lucy Wright, Owen Yang, and Heather Young.

The following NHS breast screening centres took part in the recruitment and breast screening follow-up for the Million Women Study: Avon, Aylesbury, Barnsley, Basingstoke, Bedfordshire & Hertfordshire, Cambridge & Huntingdon, Chelmsford & Colchester, Chester, Cornwall, Crewe, Cumbria, Doncaster, Dorset, East Berkshire, East Cheshire, East Devon, East of Scotland, East Suffolk, East Sussex, Gateshead, Gloucestershire, Great Yarmouth, Hereford & Worcester, Kent (Canterbury, Rochester, Maidstone), Kings Lynn, Leicestershire, Liverpool, Manchester, Milton Keynes, Newcastle, North Birmingham, North East Scotland, North Lancashire, North Middlesex, North Nottingham, North of Scotland, North Tees, North Yorkshire, Nottingham, Oxford, Portsmouth, Rotherham, Sheffield, Shropshire, Somerset, South Birmingham, South East Scotland, South East Staffordshire, South Derbyshire, South Essex, South Lancashire, South West Scotland, Surrey, Warrington Halton St Helens & Knowsley, Warwickshire Solihull & Coventry, West Berkshire, West Devon, West London, West Suffolk, West Sussex, Wiltshire, Winchester, Wirral and Wycombe.
